# Drivers of fish choice: an exploratory analysis in Mediterranean countries

**DOI:** 10.1186/s40100-022-00237-4

**Published:** 2022-11-05

**Authors:** A. Saidi, G. Sacchi, C. Cavallo, G. Cicia, R. Di Monaco, S. Puleo, T. Del Giudice

**Affiliations:** 1 Department of Agricultural Sciences, University of Naples Federico II, Via Università 100, 80055 Portici, Naples, Italy; 2 Faculty of Science and Technology, Free University of Bozen-Bolzano, Piazza Università 5, 39100 Bolzano, Italy

**Keywords:** Fish consumer behaviour, Developing countries, Mediterranean area, Focus groups

## Abstract

Fish is an important source of healthy proteins and an important economic sector in Mediterranean countries. Despite the wealth of knowledge acquired in Western countries, a gap has been found in studies in developing countries, as in the Mediterranean southern shore. Therefore, we aimed to investigate consumers’ perceptions of finfish attributes, with qualitative tools as focus groups, given the exploratory nature of the research. The focus groups have been held in Italy, Lebanon, Spain, and Tunisia; in each country, one was held in seaside areas and one in inland areas, in order to control for the availability of fish that shapes consumers’ evaluations and expectations. The focus groups have been analysed through content and semantic analyses. Results of the study yielded main themes recurring in the discussions that have been categorized along such dimensions: (1) definition of fish products; (2) context; (3) search attributes; (4) experience attributes; and (5) credence attributes. Among attributes, the ones mostly guiding consumers’ choices seem to be freshness and fish species, which are used as proxies for quality and sensory attributes. Most of the respondents preferred delicate white fish, while some exceptions were found in Tunisian respondents preferring blue fish and they also were the only ones who were not looking for convenient and already cleaned products. Trust also represented a critical element in guiding the decisions of consumers: with a lack of trust, consumers deviate from preferring local products, as noticeable especially in Lebanese respondents’ opinions. Credence attributes such as animal welfare and sustainability received a minor attention from all the respondents.

## Introduction

Fish has always been of great importance not only for the economic implications in both developed and developing countries, but also a vital source of nutrition for humans (Rimm 2006; Ruxton 2011). In particular, fish has numerous virtues that make it a desirable component of a balanced diet (Thilsted et al. 2016).

The popularization of extant eating trends such as veganism, vegetarianism, and pescetarianism, along with the ongoing series of food scandals and the increase in health and nutrition concerns among people, has fuelled the reshaping of the human diet towards substituting meat with fish (Pennings et al. 2002; Rosenfeld and Tomiyama 2019; Tilman and Clark 2014; Yamoah and Yewson 2014; Yeung and Morris 2006). Furthermore, the globalization of both food markets and supply chains has been of major importance in changing people’s habits, causing a shift in consumer demand from domestic to global goods. Global population growth and the resulting increase in food demand, as well as overfishing of several key marine stocks, have affected both the supply of and demand for food and fish (FAO 2020; Hanus 2018).

In general terms, consumers acquire a particular food or service to meet their perceived needs (Agyekum et al. 2015). However, the choice of a product capable to meet specific requirements depends also on the consumer’s perception of quality and cultural background (Emilien et al. 2017; James 2004) which may be perceived differently from one consumer to another (Agyekum et al. 2015). Indeed, consumers deal with food decisions (Emilien et al. 2017): in this mechanism, both intrinsic and extrinsic cues shape consumers’ choices. The most known are: sensory characteristics, nutritional values, health aspects, price and value for money, convenience, availability and seasonality, geographical origin, production method (wild vs farmed), and product form (fresh, frozen, processed, and other) (Claret et al. 2014; Gaviglio et al. 2014; Gaviglio and Demartini 2009; Grunert 2005).

Particularly, consumers’ choices and procurement of fish are driven by a range of products, consumer traits, or situational attributes (Carlucci et al. 2015; Gifford 2002; Köster 2009). Previous studies have emphasized the effect of finfish traits on consumer choice, whether addressing contextual factors, search, experience, or credence attributes (Claret et al. 2014; English et al. 2004; Galati et al. 2022; Gaviglio et al. 2014; Gaviglio and Demartini 2009; Giacomarra et al. 2021; Grunert 2005). Maesano et al. (2020), Vitale et al. (2017), and Cantillo et al. (2020) provided recent reviews on the impact of seafood features on customer preferences and decision-making processes. However, previous research fails to provide an overall insight regarding consumer preferences as they tend to focus on specific fish traits individually (Maesano et al. 2020; Mulazzani et al. 2021; Vitale et al. 2017), thus it is needed a focus on fish as a whole to explore the main drivers behind consumer decision-making process. In addition, most previous studies were based on developed countries, with a particular focus on European Countries, with a consistent shortage of investigations in developing countries, thereby contributing to a partial view of consumer behaviour (Prato and Biandolino 2015). In fact, the share of developing countries in total fishery exports has been about 54% by value and 61% by quantity (live weight equivalent) in 2019 (FAO 2021). Although fish consumption per capita was higher in developed countries (FAO 2020), this food helps to fight against malnutrition, and it is a major generator of economic activity and employment, since it is a major contributor to domestic food security in less developed countries (Paquotte and Lem 2008; Prato and Biandolino 2015). Consequently, it is crucial to understand the main drivers of fish consumption in both developing and developed countries to better plan the population needs and preferences and satisfy consumers’ requirements in terms of fish intake.

On these premises, the overall objective of the present study is to shed light on how fish characteristics may influence preferences and decision-making.

The focus is on the Mediterranean basin, including less developed countries, adding some new insights to the current scientific debate.

A qualitative analysis involving focus groups method has been applied in four countries of the Mediterranean area, namely Italy, Lebanon, Spain, and Tunisia, with the purpose of answering to the following research questions:

- RQ1. How attributes of the product influence consumer’s preferences in selected countries?
- RQ2. How availability influences the perceptions between inland and seaside residents within each country?

The remainder of the paper is organized as follows: “Theoretical framework” section provides an overview of the theoretical framework at the basis of the research; it then will go into presenting the methodology and the data analysis in “Methodology” section. In “Results” section, the results arising from the content analysis of the focus groups are reported, while “Discussion” section provides a discussion of the results obtained by the content analysis of the focus groups conducted and, in “Conclusions” section, the main conclusions are drawn.

## Theoretical framework

Food quality is a central issue in today’s food economics (Grunert 2005). As posited by Lancaster (1966), or Molnár (1995), food quality is the assemblage of the effect of attributes which determine the product’s performance, are in dynamic interrelation, and influence the consumer in accepting the product.

Consumers use a set of factors to guide them throughout their decision-making process. These cues are not only numerous, but dynamic and changing over time and place (Devine et al. 1998; Kopetz et al. 2012). Economic theory on product quality makes a major distinction between search, experience, and credence characteristics (Darby and Karni 1973; Nelson 1970). Search characteristics are described as product characteristics that can easily be evaluated and compared by a consumer before purchasing the product (Kenyon and Sen 2012). Experience characteristics are product attributes that can only be evaluated after a product has been purchased and used (Oude Ophuis and Van Trijp 1995). Credence traits, on the other hand, are product characteristics that cannot be recognized even after the product has been purchased and consumed (Darby and Karni 1973). The distinction between search, experience and credence characteristics is crucial in understanding subjective quality perception (Darby and Karni 1973; Nelson 1970). Therefore, this categorization will be used to illustrate the opinion of consumers as disclosed in the focus groups. In addition, the dietary habits of the population in different regions of the world have been determined mainly by the availability and local practices (Shashikanth and Somashekar 2020). In general, the choice set always influences how choices take place (Vecchio and Cavallo 2019), and this is particularly true in fish choice (Thong and Olsen 2012). The main pattern characterizing fish availability is linked to proximity to the seaside, where people living nearby the sea generally have a higher fish consumption compared to inland residents (Bose and Brown 2008; Verbeke and Vackier 2005). Therefore, in this study, we will explore consumers’ opinions by splitting the sample into two tiers, according to either coastal or inland residence in different countries: Italy, Lebanon, Spain, and Tunisia for better representativeness of fish consumption (Olsen 2001; Samaniego-Vaesken et al. 2018).

Therefore, our analysis is structured as follows:

1. *Definition of fish products* Some debate originated on which products were eligible for discussion when talking about fish products.
2. *Context* Some contextual factors need to be specified, being availability the reason to split into two our focus groups and trust a factor that hampers/enhances the effect of each attribute.
3. *Search attributes* The attributes that are available to the consumer at the time of purchase.
4. *Experience attributes* The attributes that can be discovered only after the trial of the product.
5. *Credence attributes* The attributes that the consumer believes the products have but can never verify by himself.

## Methodology

### Procedure

Focus groups interviews were chosen as they are more useful for exploratory research (Cyr 2016; Morgan 1998; Smithson 2000; Wilkinson 1999). In fact, without adequate and structured knowledge is not possible to set a quantitative research analysis, in which specific research questions guide the investigation. In this case, we first acknowledged the lack of research in the Mediterranean area on fish consumer behaviour.

In focus groups interviews, the social dimension in terms of the participants’ interactions is added compared to individual interviews (Wong 2008). Participants are encouraged to exchange thoughts and opinions on each other's points of view (Kitzinger 2006). Therefore, a thorough insight into what moves and inspires the target group can be collected.

**Fig. 1:**
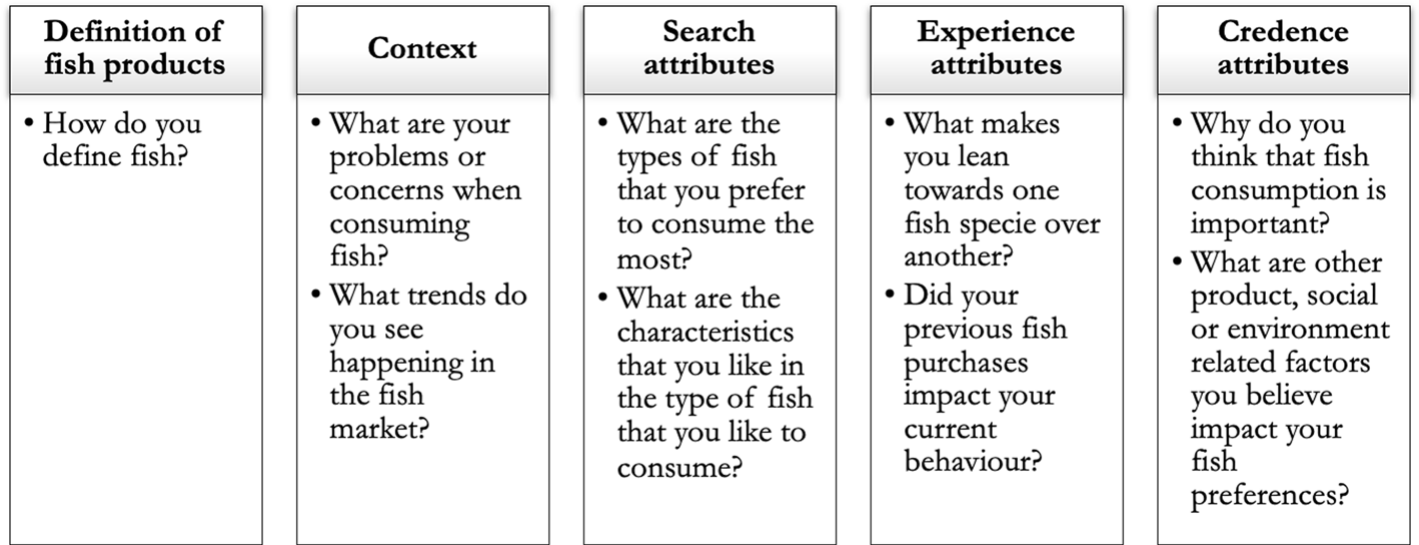
Thematic categorization of focus groups

The first step has been the gathering of semi-structured open questions in a manual. Following the theoretical framework, questions were grouped into three themes: (1) search; (2) experience attributes; and (3) credence attributes. Figure 1 shows an overview of the questions administered during the focus groups.

The focus group protocol was then tested during a pilot discussion in Tunisia in August 2020 and thereafter validated. Following, two focus groups per country (Italy, Spain, Tunisia and Lebanon) for a total of eight sessions were held from September 2020 until March 2021. The countries were chosen with the aim of representing the whole Mediterranean area, of much importance for its diet (Prato and Biandolino 2015). In each country, one session was held with consumers living near the seaside and the second one with residents of internal areas. Hereinafter, participants who live near the seaside are referred to as “*seaside residents”* while those living in internal areas are referred to as *“internal residents”*. Table 1 provides the research procedure used for this study.

**Table 1:** Procedure adopted for the research

Study propositions	To improve knowledge of the current preferences on fish consumption in the Mediterranean basin To understand fish characteristics and their influence on consumer decision-making
Research questions	RQ1. How attributes of the product influence consumer’s preferences? RQ2. How availability influences the perceptions between inland and seaside residents in the countries investigated?
Units of analysis	Four countries of the Mediterranean area: Italy Lebanon Spain Tunisia
Linking data to propositions	1. Definition of fish products 2. How the context influences preferences for fish products 3. Analysis of *search* attributes 4. Analysis of *experience* attributes 5. Analysis of *credence* attributes
Method of analysis	Focus groups: two per areas (seaside/inland) per each country, tot.: 8 focus groups
Criteria for interpreting the study’s findings	Content analysis: Word-count analysis Text coding Aggregation of similar and related topics Semantic analysis: Analysis of co-occurrences Score assignment to different cues
Timing	September 2020–March 2021

### Selection of participants

To be part of the sample, participants have to comply with the following requirements: being over 18 years old, partially, or totally responsible for the household grocery (specifically fish purchases), and balanced between living either from the seaside or inland. The recruitment of the participants was conducted in a way that respects the above-mentioned conditions to capture fish traits among people who confront fish pre- and post-consumption. Afterwards, focus groups were conducted in both inland and seaside zones, motivated not only by differences in fish availability, but also by diverse dietary patterns of the people, as those living near the sea tend to incorporate more fish in their diet.

The sample consisted of 77 participants: 27% were from Italy, 17% from Lebanon, 23% from Spain, and 32% from Tunisia. The northern Mediterranean countries were represented by Italy and Spain, while the southern Mediterranean countries were represented by Tunisia and Lebanon. 47% were male and 53% female; 45% were from internal areas, while 55% lived nearby the seaside. Respondents between 18 and 29 were the largest share of the total sample (26%), the 32% did not specify their age (Table 2). The absence of age specification has been accepted for privacy purposes.

**Table 2:** Description of the sample

Variables	No. of participants	Percentage
Country		
Italy	21	27
Lebanon	13	17
Spain	18	23
Tunisia	25	32
Geographical area		
Inland	35	45
Seaside	42	55
Gender		
Female	41	53
Male	36	47
Age		
18–29	20	26
30–40	12	16
41–50	13	17
51–60	5	6
Over 60	2	3
Unspecified	25	32
Total	77	100

### Data analysis

All focus groups discussions were audio-taped, video-registered, and word-by-word transcribed. Discussions were conducted by native speakers of Arabic, Italian, and Spanish, and afterwards, all transcriptions were translated into English and used as input for the content and semantic analysis purpose.

The content analysis is a systematic and descriptive method used to analyse words or phrases within a wider range of spoken or written communication. It uses units of analysis extrapolated from the messages that coincide with the significant elements of the text. Content analysis can have different extensions and semantic complexity ranging from single words to full texts.

We have also followed the grounded theory principles (i.e. the collection of theories suggested by patterns found in data) and deductive methods (i.e. the process of reasoning from certain laws, principles, or the analysis of facts) with an emphasis on emergent themes (Charmaz 2011).

As a first step, we performed with the software NVivo 12 the word-count analysis of each transcription. The word count was conducted separately by the authors to identify the most recurrent words and phrases and then the most recurrent themes were coded based on topic similarities. For consistency reasons, we have also applied a coding following the “classic approach” otherwise known as the “scissor-and-sort” technique. In more detail, the printed transcripts were cut up grouping similar quotes and then assigning the codes to the quotes (Braun and Clarke 2006; Billups 2003). Once the codes were established, they were put together into memos and the memos were subsumed into themes. The consistency, coherence, and distinctiveness of the themes were confronted with those that emerged by the NVivo analysis and double-checked by the researchers involved in the study who operated separately and compared their evaluations only at the end of the process.

As a second step, based on the recurrent words/concepts, we performed a semantic analysis of the topics identified. Indeed, this method allows to explore the relationships between identified themes; in this case, what it seeks is the meaning derived from the relationships between concepts in the text. A list of cues was consequently agreed upon among researchers and scales were built based on the relevance of the words and topics to the attributes that determine consumers’ choice of finfish. When assigning scores, the neutral perception of the cues was also considered (i.e. when a certain attribute was mentioned several times but in phrases that stated its low importance) without influencing the assignment of the scores. For instance, if a participant referred to the topic “price” in a neutral way (“*I’m not sensitive to the price of the product while to me it is important its availability within the local market*…”), even if in the word-count analysis the statement was added to the topics “price”, “availability”, and “local market”, during the semantic analysis we avoided to consider the recurrence of “price” and we considered only the “availability” and “local market” ones.

## Results

**Fig. 2:**
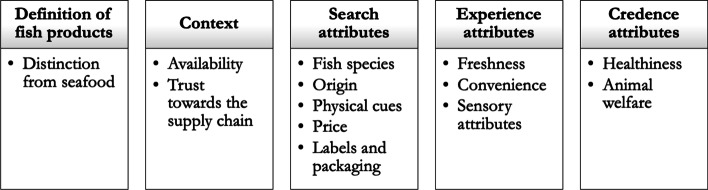
Factors influencing fish consumption and consumers’ choice

The analysis of focus groups yielded the definition of a set of attributes that respondents highlighted as important for their fish choices. In Fig. 2, main insights are summarized, according to the starting thematic scheme. In the following paragraphs, more detail will be given about how most recurrent themes occurred during the discussion and how each attribute has been intended by the discussant and whether there have been differences between seaside-inland residents or per country of origin.

Following, according to the results of content and semantic analysis, the scores assigned to the elements determining fish choices have been plotted in graphs and differences between groups of the same countries are discussed.

In the following sections, results of the analysis are discussed considering some memorable sentences that arisen in the FGs which allow for further justification of our findings.

### Definition of fish products

Overall, most respondents did not have a clear definition of fish and were unable to distinguish between all sea goods. Many respondents were indeed unable to distinguish between finfish and shellfish. This point is well summarized by one participant who stated: “*for me, everything that lives underneath water is fish. Then if the experts want to classify it into different categories, that is their choice*” (Tunisian participant).

Consumers from South Mediterranean countries were aware of the distinctions between different fish categories according to the appearance. Some respondents from Northern Mediterranean countries are also able to differentiate between finfish and shellfish using physical cues as claimed, for instance, by a Spanish participant: “*fish is everything that has to do with animals that comes out of the sea, I would not consider seafood as fish as the body structure is quite different*”. Nevertheless, the overall tendency was to consider all marine commodities as fish.

### Context

#### Availability

Availability represents a key element in fish consumer behaviour. Even with global supply chains, seaside and inland residents have a different choice set when buying fish (Misir et al. 2015). Therefore, this motivated us to conduct separate focus groups for seaside and inland residents.

This was confirmed by the first analysis of our focus groups discussions: all participants agreed on fish availability being pivotal for their choices: “*the fact that I live far from the coast and the lack of ports significantly reduces the frequency of fish consumption*” (Tunisian participant). Most participants’ decision was actually based upon “*what is available and the advice of the fishmonger*” (Italian respondent).

Some inland residents pointed out to reduce their fish consumption due to a scarcity of fish species sold at their available sale channels: “*the lack of taste characteristics of fish similar to swordfish and salmon, reduced fish consumption*” (Tunisian participant). This was also valid for all other inland residents, participants had problems finding fish that met their requisitions in terms of freshness, quality, safety, and price. In contrast, seaside residents did not refer to availability as a driving factor in their decision-making process.

The availability of fish also has an impact at the time of buying. All respondents agreed on making their choice within the shop/market. Most respondents stated that “*they never buy what they decide to get prior to going to the fishmonger”* (Italian participant), and that their choice is dependent on what is available: “*I generally go out of the house to buy grouper, but that changes the moment that I arrive at the local market where the offer doesn’t correspond to my needs*” (Lebanese participant). Others combined availability with intrinsic and extrinsic product features as appearance and price to make their choice. *I usually choose what I like the most, what is most appetising, and what looks the best from the available options at the local market*” (Spanish participant).

Lastly, the availability can impact the familiarity with the product, and hence the habit to include it in the diet and the ability to cook. In fact, in Tunisia in particular, internal respondents felt their knowledge of fish to be restricted since they live far from the sea, as opposed to those who live in coastal areas, where fish is a staple of the diet since childhood.

#### Trust towards the supply chain

The effect that each attribute can play in the consumer’s mind is believed to be mediated by trust (Giampietri et al. 2018). Furthermore, fish is a food category that is particularly susceptible to food safety issues and food scandals (Visciano and Schirone 2021). Therefore, we collected the trust opinions and concerns expressed by the participants in the FGs.

Respondents from Tunisia and Lebanon did not have any trust in fishmongers and industries on product information. The perceived lack of transparency regarding fish supply chain makes consumers lose their control over the origin and production method of sea goods: “*I would love to know from where that fish came exactly. However, this kind of information is never present and even if it is, you can never be sure if it is true or not*” (Tunisian participant). Therefore, buying frozen fish from foreign brands is seen as a solution, and their traceability information is considered more trustful.

Italian and Spanish participants preferred to buy local and, overall, showed more trust in fishmongers. Specifically, Spanish respondents felt reassured by the fish markets regulating organizations. One of them claimed in particular that* “there are organisations that do their job very well in protecting consumers. So, we really must lower our guard*”.

### Search attributes

#### Fish species

Tunisian respondents displayed various preferences for fish species depending on their geographical location. There was consensus among internal residents regarding preferred fish species: sardines, mackerel, tuna, and sea bass being the main choice. Furthermore, participants claimed to consume also other species such as sea bream, bluefish, red pandora, red mullet, and dentex whenever possible. Others also eat salmon, swordfish, and grouper occasionally, as they are considered among the premium species in the Tunisian market. While most seaside residents prefer to eat saupe and dolphinfish^1^ even if these species are difficult to be found in local markets, followed by red mullet, sea bream and sea bass. White fish species are preferred by most respondents. Finally, a small minority showed a preference for blue fish, specifically sardines and bogue fish.

In Lebanon, all participants prefer to eat salmon, sea bream, tuna, common pandora, grouper, and swordfish. Generally, all white fish species tend to be preferred.

In Italy, differences have been found between seaside and internal residents. While inland residents preferred blue fish species such as anchovies, salmon, and cod followed by sea bream, swordfish, red mullet, salted cod, and plaice; seaside residents preferred mainly sea bass and cod followed by salmon, and swordfish. Italian participants from internal areas showed a huge interest in the consumption of salted cod, especially due to its availability all year round, shelf life, and also the fact that it is an ingredient present in many easy-to-cook recipes.

Spanish participants’ preferences for fish species were relatively homogenous and they were mainly directed towards salmon (smoked or fresh), tuna (fresh or canned), cod (fresh or frozen), sea bass (fresh or frozen), sole, sea bream (fresh), and swordfish (fresh).

#### Origin

The origin of fish is a crucial aspect linked to consumers’ choice. Participants from Tunisia, Italy, and Spain prefer to eat local while Lebanese and inland Tunisian respondents leaned towards imported fish because of the more stringent regulations they rely upon. A particular emphasis on origin is found for pre-packed sea goods that carry this information clearly on the label.

The origin can also be intended in terms of caught or farmed fish. It is not a determining factor amongst Northern Mediterranean interviewees as most of them seem not to pay attention to whether fish is wildly caught or farmed. On the other hand, Southern Mediterranean respondents showed some preferences for caught fish as well expressed by a Lebanese participant: “*I would like to consume more locally caught fish to support fishmongers and local economy*”. Furthermore, wild-caught fish was considered tastier and less smelly compared to the farmed alternative. Lebanese consumers are concerned about the seawater pollution as “*fishmongers do not care if the product that they are catching is polluted or not*” (Lebanese participant). Few participants agreed that farmed fish is better controlled and helps reduce the overexploitation of marine resources. Finally, other respondents stopped purchasing farmed fish for its high fat content.

#### Physical cues

Respondents from the four selected countries choose fish based on specific physical characteristics, especially those that are traditionally used to infer fish freshness.

Tunisian respondents mainly claimed to choose fish based on size, with a preference for medium to large fish. Small fish species were associated with an unpleasant eating experience due to the lack of meat and the presence of little spines and fishbones. Consumers also considered the general appearance, brightness of the skin and eyes, bloody gills, evidence of bleeding, and firmness of the flesh. For instance, a Tunisian participant stated: “*the brighter the eyes, skin and the redder the gills are, the fresher the fish is*”.

In Lebanon, people also leaned toward large fish and relied on the general appearance such as the absence of spines and fishbones, brightness of the eyes and firmness of the meat to select one fish species over another.

Italian respondents also used the general appearance, the vividness of the eyes, the absence of spines and fishbones, and the firmness of the meat to make their decision choice. No differences according to age and living area were noted regarding the impact of physical features on consumers’ choice. Nonetheless, Italian male participants were more likely to base their decisions on physical traits.

In Spain, most respondents had limited knowledge of fish regardless of their living area. Nonetheless, the overall appearance and the size were the main features that helped consumers when making their purchase. The smoothness, brightness of the eyes and skin and the absence of the spines were used by a few Spanish respondents when buying sea goods.

#### Price

Price is the main attribute that guides consumers’ choices. In the case of fish, it is seen as a constraint as “*fish is usually more expensive than other types of meat*” (Tunisian participant).

Fish is perceived as luxury good by respondents from Tunisia, who reported including fish at least once a week for health reasons, even if expensive.

Similarly in Lebanon, while the ongoing Covid-19 pandemic and the country's financial crisis have helped to lower the price discrepancies between fish and other types of meat, Lebanese participants still perceive fish as an expensive food.

In Italy, the price was more relevant for seaside residents compared to inland ones. Inland residents were more concerned with other factors such as freshness, availability, and seasonality, meaning that they were less price sensitive when the product meets their requirements.

In Spain, only inland residents reported it as a barrier as expressed by one respondent “*when I go buying fish, I try to balance my purchase, mixing expensive and cheap options*”.

Price is also used as a signal of quality. Few participants linked a cheaper fish price with a lower quality: “*I really care about the freshness and the price-quality ratio*” (Tunisian participant), “*the price and quality ratio are the biggest determining factor when it comes to buying fish*” (Spanish participant).

#### Labels and packaging

In Tunisia, the majority of the respondents reported their preference for wild-caught fish, unpacked and unlabelled. Some exceptions were for canned tuna by famous brands such as El Manar or Sidi Daoud. Seaside residents showed a strong preference for local brands, stating that they “*will never buy fish that has been imported from another country*”. While inland residents preferred imported brands as they perceived more transparency and better quality.

While Italians stated to have no preferences in terms of brands and that, in the case of packed fish, they are “*mainly guided by promotions*”. They did not show any interest or preference regarding the packaging of sea goods, as the respondents were from Tunisia.

While respondents from Lebanon and Spain showed a preference for a particular type of packaging: simple, transparent, and soft colours that remind the colour of the sea. Also pressurized and individually packaged slices were valued as they reassure the product quality. The presence of water mist on the packaging represented a barrier to some participants from buying sea goods as it evoked poor quality and enabled them to clearly see the product inside. Spanish respondents also reported the importance of labels to infer good quality and a more flavourful fish.

The preference for foreign fish in Lebanon is reinforced by the analogous preference for foreign brands of fish, as perceived as more compliant with food regulations.

### Experience attributes

#### Freshness

Freshness has been recorded as the most important aspect of consumers’ choice. Freshness is so vital that some participants opt to buy frozen sea goods instead of fresh fish when local markets cannot meet their expectations.

In many cases the value of freshness is seen as an indicator of the overall quality of the product- For example, some respondents linked freshness to nutritional value as fresh fish was considered more nutritious than the frozen or pre-packaged alternatives.

Freshness cannot be ascertained at the moment of purchase in many cases; therefore, some cues like smell or visual peculiarities are used as signs of freshness.

While for other respondents it is an experience attribute that is discovered at the time of eating with texture: “*when fish is not fresh, I get an itchy sensation in my mouth, which is not the case of fresh fish that usually has a smooth texture and is very moist*” (Tunisian participant). Also, seasonality is used as a cue for freshness, and it is linked with tendentially cheaper prices. In particular, an Italian respondent stated that (s)he knew *“the appropriate periods of consumption for particular fish species*”, while a Tunisian participant stated that (s)he tends “*to buy species according to the fishing season for several reasons, most importantly to have a fresh product*”. It can also be used as a cue for good taste*: “any fish that is caught in its season is delicious”* (Tunisian participant). On the contrary, Lebanese and Spanish respondents did not consider fish seasonality in their discussions.

#### Convenience

Convenience is an important feature for the totality of the sample to the extent of driving the consumption of one or another species or avoiding entirely the purchase (i.e. consumption of sardines is generally avoided as their preparation is perceived as time-consuming and effort-taking).

Italian and Spanish participants considered fish preparation very time-consuming and not well adaptable to many recipes. For this reason, some participants stated to prefer eating frozen fish, as it must be cooked without any additional cleaning or preparation.

Cleaning fish is tendentially avoided by all respondents, but an exception was found in seaside Tunisian residents, they enjoyed cleaning fish as it evoked memories of their childhood. One of them stated in particular that “*I used to watch my mom clean fish, so I grew up watching her do it and I always wanted to imitate her when I get married*”. Some inland Tunisian respondents considered a barrier to fish consumption the lack of culinary skills for fish-based dishes. A demonstration of this aspect is well expressed by a Tunisian respondent who claimed that “*it all rolls back to the culinary habits linked mainly to the geography, I as a well as a lot of people her in Tebourba prefer to buy lamb and chicken meat because it gives us a larger option of plates to prepare and one do not know how to prepare a lot of fish-based dishes*”.

Some Italian respondents stated to avoid convenience problems by purchasing fish that was already cleaned by their local fishmonger: “*the cleaning process is the thing that I hate the most. So, my local fishmonger cleans it and bring it to my house so it is a very nice service that I will not be able to get it somewhere else*”. But this service does not appear to be popular in most of the sales channels of other countries; therefore, the majority of respondents from Lebanon, Spain and Tunisia do not rely on it.

#### Sensory attributes

Sensory attributes are somehow considered important cues for fish consumption, especially taste and smell. All Lebanese, Italian, and Spanish seaside residents prefer neutral taste and a non-slimy texture. Only a few respondents preferred the salty flavour, that they associated with wild-caught fish. Furthermore, the smell is a valued attribute at both the time of purchase and the time of consumption as a strong unpleasant smell can be a significant barrier for all respondents. Tunisian respondents reported being a major barrier to eating blue fish species and used this cue to infer lower freshness. Similarly, some inland Italian residents stated to avoid anchovies for their strong smell.

Lebanese respondents considered fish to be naturally a smelly food but, instead, they reported paying attention to the smell of the environment as reported by one of them: “*I know that fish has a smell naturally, but the marketplace doesn’t have to smell horrible*”. While some Spanish respondents resented the fish smell getting on their hands, and they even avoided patronizing fish because of it.

Finally, the general appearance of the product in the market or within the shops is also claimed to be an important aspect guiding consumer’s choice. The organization of fish stalls and the overall cleanliness of the selling place made Lebanese participants at ease when buying fish: “*the overall appearance of the environment is what really draws my attention (the cleanliness of the shop, the lighting of the shop and even the fishmonger)*”. Whereas respondents from Italy, Spain, and Tunisia focused their attention mainly on the products rather than the setting in which they are traded.

### Credence attributes

#### Healthiness

The nutritional value is one of the main drivers of fish consumption among all participants, as fish is believed to contribute strongly to a healthy diet. Indeed, most of them agreed on fish being an important source of protein, omega 3 content, and oligo-elements.

This can be more important in the light of meat restrictions increasingly popular among consumers. In fact, for some respondents, especially from Spain and Lebanon, it represented the sole alternative to eating high biological value proteins: “*I don't eat red meat, so one of my main sources of protein is fish*” (Spanish participant). While Italian and Tunisian respondents appeared to be less restrictive about food sources.

Even participants loving red meat (beef and lamb), perceived a higher nutritional value in fish: “*I still prefer red meat rather than fish even though fish has a higher nutritional value which makes me include it in my diet*” (Lebanese participant). The awareness of the health content of fish was higher among seaside residents, while inland residents across all countries neglected more the nutritional value of fish.

For some consumers, the choice of fish is motivated by food safety issues: “*with all the scandals happening consecutively for the other types of meat like chicken and beef, I started to become more aware of what I put in my body and leaned more towards fish*” (Tunisian participant). Some beliefs are valid only for some species as blue fish is perceived as more beneficial to health while large fish were considered to contain more heavy metals and to be sources of contamination compared to medium or small fish species: “*I prefer to eat sardines over Red Pandora because from what I know, sardines have a higher omega 3 intake*” (Tunisian participant).

Lebanese respondents considered the lack of environmental regulations in the country a main driver of fish pollution as most industries discharge wastewater, full of chemical residues, into the sea, endangering the health of people. This outcome was found to be a major barrier to consuming local fish. Lebanese participants also reported some concern for the healthiness of fish due to the content of pollutants.

#### Animal welfare and environmentally friendly aspects

Many participants did not mention animal welfare or environmental sustainability in their fish choice since they perceive a very low impact on the environment from their consumption behaviour. This perception has been well explained by an Italian participant who stated that: “*I do not think that I can have that much impact on the environment. So, when I buy fish or any other product, I do not think of the repercussions of my behaviour on the environment*”.

Lebanese respondents were the most concerned about sustainability believing that the available marine resources are not able to meet the population’s needs and therefore they expressed the need for more regulations for protecting the environment: “*using very small fillets to catch as much fish as they can contributes significantly to the reduction of the natural available stocks of fish*” (Lebanese participant).

Some respondents, mainly seaside residents, did show concern about the overexploitation of marine resources, the pollution of the environment and the consumption of endangered species. Some Italian, Lebanese, and Tunisian respondents emphasized the need of “*more laws about the modalities and methods of fishing to be able to ensure a sustainable fishing supply system to consumers*” (Italian participant).

Tunisian respondents reported aquaculture as a viable way to protect some fish species, but showed also a concern for its sustainability, due to the use of chemicals.

### Comparison between Inland and Seaside respondents

According to the content analysis, we collected some scores for each element that we included in the model to explain respondents’ behaviour towards fish products across countries in the Mediterranean basin. The scores have been split into the groups in which we divided the focus groups: the seaside and the inland residents. Following we report the main issues that emerged during the discussions.

**Fig. 3:**
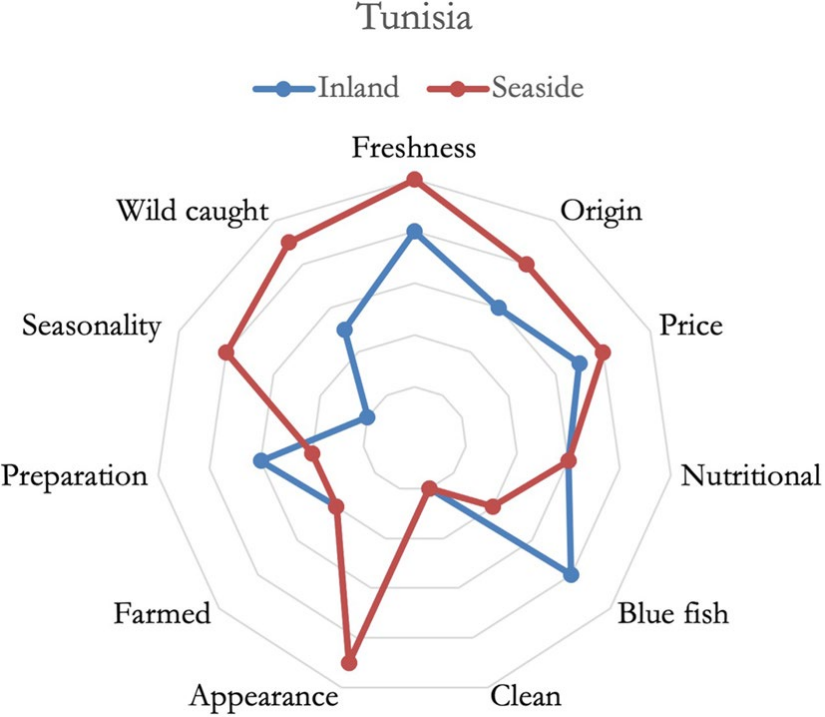
Importance of fish attributes in defining consumers’ preferences, Tunisia

In Tunisia, as reported in Fig. 3, the respondents based their fish purchases mainly on price, freshness, and origin. Seaside residents placed more importance on origin, wild-caught fish, but also valued seasonality, instead inland residents placed more importance on blue fish species and convenience. Tendentially, context and credence attributes were slightly influential in consumers’ choices.

**Fig. 4:**
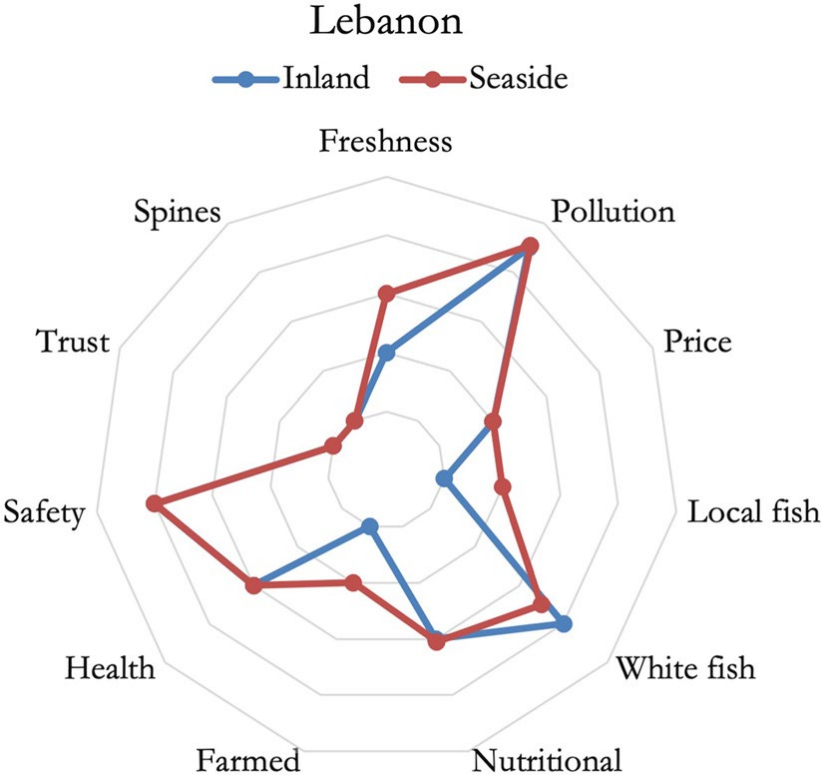
Importance of fish attributes in defining consumer's preferences, Lebanon

In Lebanon, as shown in Fig. 4, no major differences were noticed between inland and seaside residents. Only the perception of farmed fish was higher for inland residents, and the importance of freshness was higher for seaside residents. In general, Lebanese respondents considered fish to be healthy and preferred white fish species without spines or bones. A serious issue regarding the trust towards the supply chain has been delineated, it emerged also during the previous analysis, and this substantially impacted the differences in perceptions between Lebanese respondents and respondents from all the other countries.

**Fig. 5:**
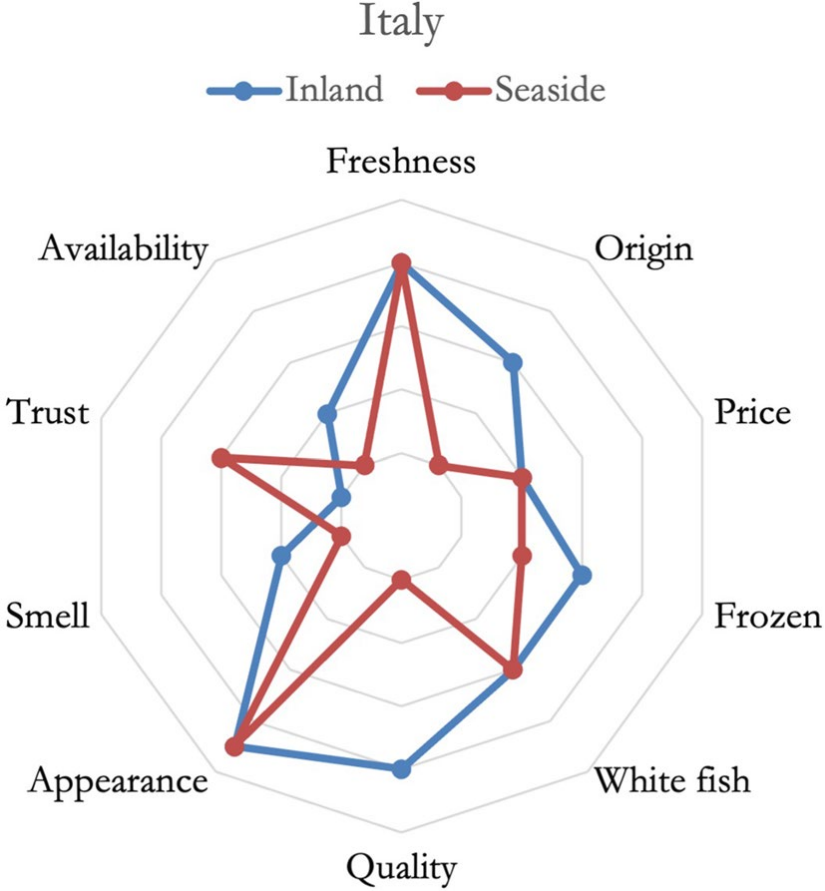
Importance of fish attributes in defining consumer's preferences, Italy

In Italy, as shown in Fig. 5, all respondents agreed on the importance of white fish species and considered freshness to be way more important than other aspects, a minor importance attached to price was constant for all respondents. The two groups had some major differences: seaside residents displayed more trust in the fish supply chain compared to others. While inland resident, lacking trust, relied more on other aspects such as the origin of the product, quality, and intrinsic aspects such as smell. Inland residents also stated to rely more on frozen fish over fresh ones for availability constraints. The content analysis also yields that credence attributes were neglected in the discussion compared to others.

**Fig. 6:**
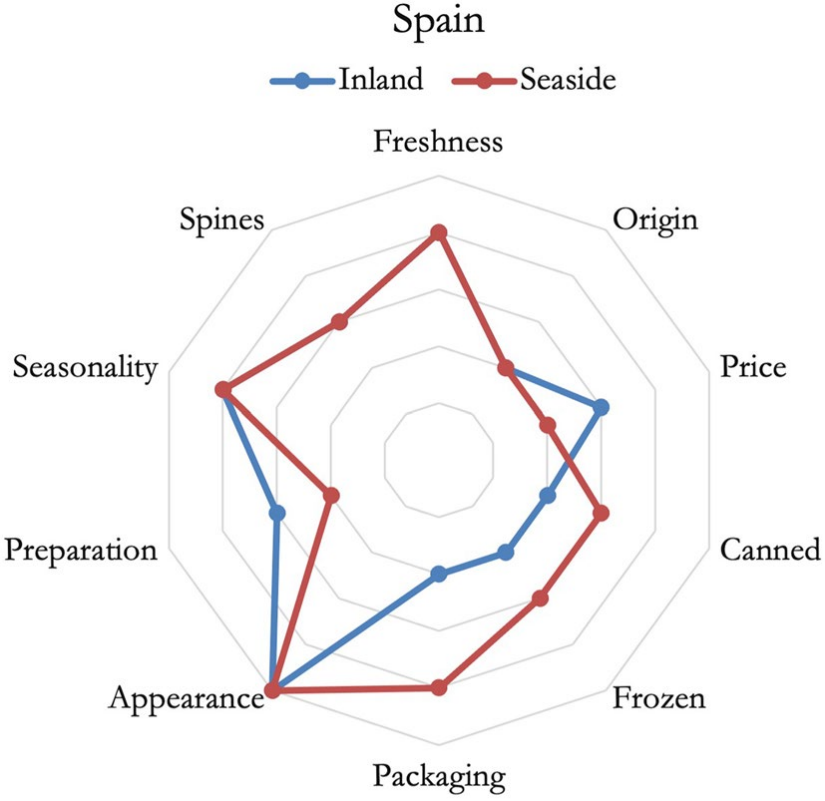
Importance of fish attributes in defining consumer's preferences*, Spain*

Lastly, as shown in Fig. 6, Spanish respondents agreed on liking fish mostly with a good appearance. Also, freshness and seasonality were deemed as important elements of choice, while origin was slightly important for all respondents. The two groups showed some differences: seaside residents highly valued the packaging of fish and the frozen form. While the inland respondents were more interested in the types of preparation that the product requires and more interested in price compared to others. The importance of credence attributes appears to be minor compared to other aspects of the product.

## Discussion

The results of the focus groups showed homogeneous results across discussions that occurred in different Mediterranean countries. Unlike other foods such as meat, wine, or cereals that can define a clear consumption pattern for food products among people from different countries, finfish is still unable to do so even though a progressive departure from the traditional Mediterranean diet is being observed mainly in younger generations (Tur et al. 2004).

The analysis of focus groups firstly indicated that decision-making of the respondents tends to occur directly within the shop/market and not before, and that availability is a substantial constraint in purchases, especially for inland residents. In this sense, we can suppose that fish purchases take place tendentially quickly with a more impulsive vs. rational decision-making style (Cacioppo et al. 1986). The importance of availability led us to split each focus groups in two: seaside and inland residents, in order to control the effect of this variable in our results. This has been already highlighted as a reason for not purchasing fish by other Authors (Hinkes and Schulze-Ehlers 2018) who found a high opt-out rate among consumers who did not like any of the options presented (Vanhonacker et al. 2010). Fish availability seems to be correlated with the area of living as those who lived in the internal area consumed fish less frequently compared to those living near the seaside where fish is generally more available and a part of people’s diet, in line with previous studies by Bose and Brown (2008), Heffler et al. (2011), and Verbeke and Vackier (2005). Furthermore, consumers who live in predominantly fish-consuming regions prefer to eat fresh fish products, in line with previous studies by Tomić et al. (2016) and Altiok et al. (2021).

Furthermore, empirical evidence has previously shown how fish decision-making traits differ among different countries (Altiok et al. 2021; López-Mas et al. 2021; Menozzi et al. 2020). In this study, while Tunisian and Lebanese respondents focused more on health and food security-related cues, Italian and Spanish respondents focused on sensory and physical attributes and convenience of use. This might be related not only to consumer’s trust in control organization, but also to the differences in dietary habits and consumers’ cultural background as already underlined in previous research (Murray et al. 2017; Zuzanna Pieniak et al. 2007; Temesi et al. 2020). In more detail, trust also appeared as critical with high levels of trust corresponding to lower attention devoted to the other aspects of the product. When trust issues were identified, respondents tended to prefer imported foods from trusted countries, and they paid particular attention to foreign quality certifications (Wu et al. 2021). Respondents from Lebanon declared to be concerned about pollution, and this impacted the perception of attributes such as freshness and local origin. It is generally assumed that the local origin of fish has been proven to raise consumers’ expectations in terms of tastiness and food safety (Maesano et al. 2020; Murray et al. 2017; Zander et al. 2018), but this case represents an exception.

Considering search attributes, the most important appeared to be fish species, origin, and price. Specifically, respondents tendentially preferred white fish, but Tunisian respondents showed a preference for blue fish, instead. This is because respondents learnt the sensory traits and the nutritional benefits belonging to each species (Lawley et al. 2012; Pohar 2011; Verbeke et al. 2007). Regarding the origin, academic literature has extensively dealt with the key role of this trait in consumer choices of fish (Cantillo et al. 2020; Giosue et al. 2018; Maesano et al. 2020; Masi et al. 2022; Murray et al. 2017; Paredes et al. 2020; Risius et al. 2017; Witkin et al. 2015) as consumers prefer to consume local fish products. This can be due to the natural tendency to ethnocentrism in food preferences that can be augmented by concern in food safety (Delong et al. 2016) and is mediated also by the evaluation assigned to the particular country, being products from emerging economies, tendentially perceived riskier (Wang et al. 2018).

Hence, certification and labelling systems might be a solution to strengthening consumer’s perception by increasing their awareness of ecological, environmental, ethical, and safety features. Eco-labels appear to be important in the context of fish because they fulfil the need of consumer to be more informed about the environmental sustainability of his nutrition (Brécard et al. 2009), and being able to pay more for these labels (Folwarczny et al. 2022), they can act as a trigger for innovations in the sector that shift the production processes towards a reduced impact on the planet and on fish stocks (Prieto-Sandova et al. 2016). However, the spread of eco-labels is a tendency that occurs mainly in developed countries and struggles to affirm in developing countries (Prieto-Sandova et al. 2016) because of the tendency of affirmed global labels in marginalizing smaller producers and producers in poorer countries; therefore, it is more likely that its success is dependent upon the initiative of NGOs that must be supported by policy actions (Ponte 2018).

A lower price is preferred by most of the respondents, in line with the economic theory; however, there are some cases in which a higher price is seen as a proxy for higher quality (Cicia et al. 2002). In the case of fish, there are some segments that prefer fresher and bigger size products above any other aspect, including price (Mitra 2020).

The appearance of the product is also important for respondents, especially in the Spanish part of the sample. In general, visual cues are used to infer the freshness, one of the strongest drivers of consumption. Therefore, those who use to inspect the product do not like the presence of packaging. The respondents reported using several sensory cues as: the brightness of the eyes and skin, red gills, texture, and light smell (López-Mas et al. 2021; Lawley et al.^2020^; Thapa et al. 2015). Generally, wild-caught fish appears to be preferred over farmed ones, apart from Lebanese inland residents. This is in line with previous studies that highlighted preferences of consumers may vary from wild-caught to farmed fish according to different parameters (Mitra 2020; Mitra et al. 2021; Wongprawmas et al. 2022).

Considering experience attributes, respondents appeared to be mostly concerned with freshness: it is used as an indicator of the overall quality of the product, and it is inferred by other available cues in the environment. We already mentioned the role of sensory cues, but also extrinsic attributes can be used, for example, origin, seasonality or texture, and mouthfeel. Convenience plays a particular role in the decision of consuming fresh fish, to the point of being a barrier in some cases (Ankamah-Yeboah et al. 2019; Cantillo et al. 2021; Carlucci et al. 2015; Pulcini et al. 2020). Italian respondents stated to purchase fresh fish only if a cleaning service is provided by the fishmonger. While an exception was represented by Tunisian respondents: they showed pleasure in the preparation and cleaning process of fish. This can be understood in terms of coproduction value, which states that convenience has origins in shifting consumer values and that individualism and self-fulfilment may conflict with traditions such as frequent family meals and a lot of time in the kitchen (Heide and Olsen 2011; Scholderer and Grunert 2005).

Lastly, we must consider the effect that credence attributes play in consumers’ decisions, they are generally more important where more wealth is available to consumers (Yang and Renwick 2019).

The healthiness of fish is an important driver of its consumption, since health concerns tend to reduce the expenditure on beef and chicken Pihlajamäki et al. (2019) and Morales and Higuchi (2020).

Previous studies were devoted to issues as animal welfare and sustainability, but in our  focus groups these elements did not appear as salient in the mind of consumers (Zander et al. 2018; Hynes et al. 2019; Jacobs et al. 2015). This can be motivated by the peculiarities of the product investigated, as already Pieniak et al. (2009) indicated that credence attributes are ranked substantially lower than search attributes in the case of fish. Another reason can be found in the saliency of short terms goals over long-term ones when the consumer is facing a purchasing occasion, which leads to an attitude–behaviour gap, for animal welfare this is particularly true (Verbeke 2009). Some authors also suggest that credence features are becoming so complex that the consumer finds it hard to process a big amount of information in a short time (Del Giudice et al. 2018; Nuttavuthisit and Thøgersen 2017).

## Conclusions

Fish is an important product in the Mediterranean area, for both national economies and consumers’ diets; therefore, it represents an interesting target market to be investigated in order to understand, in a deeper way, the opinion of consumers. Despite a wide array of research conducted in Western countries, structured knowledge still lacks in developing countries, such as the ones of the southern Mediterranean shore.

Therefore, this study leveraged qualitative analysis to undertake an exploratory analysis of the consumers’ points of view on finfish in four countries: Italy, Lebanon, Spain, and Tunisia. This seemed the most fitting method for investigating topics with no abundant previous knowledge. To this purpose, focus groups have been conducted in the selected countries, in each country, in order to control for the different availability of finfish products, two groups of consumers have been analysed: seaside and inland residents.

The focus groups have been analysed with a qualitative two-step research that yielded some interesting results. Going back to the initial research questions, we can then provide some answers.

The first issue that has been investigated was related to the understanding of how different attributes of the product influence consumers’ preferences in selected countries. Tunisian respondents appeared to be the only ones valuing blue fish, while all others preferred the characteristics of white fish. Wild-caught fish is preferred by most of the respondents with few exceptions found in some respondents from Southern Mediterranean. Lebanese respondents stated to be slightly price sensitive and preferring foreign frozen products and foreign certifications as concerned by local water pollution and, therefore, feared local fish products. Spanish respondents are the ones most preferring canned, frozen, and pre-packed fish and especially concerned about its convenience. While Italian respondents stated to be mostly concerned by the freshness of the product embedding all other quality attributes and are the respondents who showed the highest level of trust towards the capacity of the supply chain in providing fresh and healthy fish.

The second issue investigated regarding the analysis of the influence of product availability on consumers’ perceptions comparing inland and seaside residents within each country. Therefore, we compared groups with different finfish availability levels. We have found that respondents from continental areas are concerned about the accessibility of finfish, and this lowers their price sensitivity (with low availability). Inland respondents are more prone to buy pre-packed and frozen fish to overcome the availability problem, sometimes they rely on peculiar forms that extend fish shelf life, as salted cod. Seaside respondents also appear to be more knowledgeable about seasonality and preparation of fish and sometimes, they are less bothered by the cleaning of fish, stating even to enjoy this activity.

In the end, respondents showed a need of reassurance on the freshness, quality, and healthiness of fish. Hence, information asymmetry reduction activities would be desirable, in terms of both augmented traceability and consumers’ education. We must also acknowledge that respondents had conflicting purchasing motivations. For example, their desire to eat better-tasting fish may compete with convenience or healthiness. In fact, fresh fish is perceived as tastier but requires a longer preparation time, whereas frozen food has a more detailed label and is boneless, but it is considered less tasty and less nutritious.

Our study provided some exploratory insights on finfish consumers’ point of view in several countries, some of them neglected by the previous literature as Tunisia and Lebanon, in which fish has a prominent role in consumers’ diets and national economy. Consumers’ preference for fish has been regarded as one of the critical factors in determining consumption. From this perspective, the government, aquaculture producers, and fish restaurant operators need detailed information on individual-level preferences for fish, and this study might enable decision-makers to have an overall idea about consumers’ preferences.

This study provides significant managerial implications for the consumption of fish within the Mediterranean area. From the estimation results, marketing managers can get useful information to design strategies to increase fish consumption, especially for internal area residents. In other words, they need to make more efforts to consumer segments with a lower probability of increasing consumption frequency by informing consumers about the sensorial, physical, and health properties of fish products. Moreover, marketers need to start teaching consumers about product attributes such as origin, production method and fish labelling systems, in order for them to be more responsible when making their purchases. Then, policymakers and intergovernmental agencies can use these results in order to coordinate the trading of fish products between northern and southern Mediterranean countries to achieve a sustainable use of marine resources. In addition, the supply chain also needs to be re-evaluated to ensure a sustainable use of marine resources and the needs of consumers to the point where fish is no longer viewed as a luxury commodity.

However, some limitations must be recognized: our sample has been based on a territorial categorization, but some deeper investigations on groups of consumers with specific socio-demographic variables would be desirable. Future research needs to be undertaken in several directions. Our study concerned the broad category of finfish, that is understood differently in those countries, with usually eaten species being very different from one another; therefore, narrowing the set of finfish species would add more actionable knowledge for the stakeholders. Some limitations are embedded in the qualitative analysis that suits exploratory analysis but has to rely on a limited number of participants that lack representativeness and do not allow for the generalization of results. In an attempt to address one of the core limitations of the current study, it would be worth analysing consumer attitudes and preferences for selected fish attributes on representative samples of consumers from Italy, Spain, Tunisia, and Lebanon to be able to generalize these results. Furthermore, a more realistic research design for a higher external validity of results is also needed. This could be obtained, for instance, by virtual shelf techniques which more closely simulate the complexity of a “real” food choice environment, with respect to qualitative research method and survey-based choice, and capture consumer variety-seeking behaviour (van Herpen et al. 2016).

## Data Availability

The data sets used and analysed during the current study are available from the corresponding author upon reasonable request.

## Footnotes

1 The dolphinfish, *Coryphaena hippurus* (Linnaeus, 1758), a migratory pelagic fish with a world distribution and a relatively fast growth (Scherbachev 1973). Also widely called dorado (not to be confused with *Salminus brasiliensis*, a freshwater fish) and dolphin.
